# The Chemical Reactivity of Anthocyanins and Its Consequences in Food Science and Nutrition

**DOI:** 10.3390/molecules23081970

**Published:** 2018-08-07

**Authors:** Olivier Dangles, Julie-Anne Fenger

**Affiliations:** University of Avignon, INRA, UMR408, 84000 Avignon, France; julie-anne.fenger@univ-avignon.fr

**Keywords:** anthocyanin, flavylium, chemistry, interactions

## Abstract

Owing to their specific pyrylium nucleus (C-ring), anthocyanins express a much richer chemical reactivity than the other flavonoid classes. For instance, anthocyanins are weak diacids, hard and soft electrophiles, nucleophiles, prone to developing π-stacking interactions, and bind hard metal ions. They also display the usual chemical properties of polyphenols, such as electron donation and affinity for proteins. In this review, these properties are revisited through a variety of examples and discussed in relation to their consequences in food and in nutrition with an emphasis on the transformations occurring upon storage or thermal treatment and on the catabolism of anthocyanins in humans, which is of critical importance for interpreting their effects on health.

## 1. Introduction

Anthocyanins are usually represented by their flavylium cation, which is actually the sole chemical species in fairly acidic aqueous solution (pH < 2). Under the pH conditions prevailing in plants, food and in the digestive tract (from pH = 2 to pH = 8), anthocyanins change to a mixture of colored and colorless forms in equilibrium through acid–base, water addition–elimination, and isomerization reactions [1,2]. Each chemical species displays specific characteristics (charge, electronic distribution, planarity, and shape) modulating its reactivity and interactions with plant or food components, such as the other phenolic compounds. This sophisticated chemistry must be understood to interpret the variety of colors expressed by anthocyanins and the color changes observed in time and to minimize the irreversible color loss signaling the chemical degradation of chromophores. The chemical reactivity of anthocyanins is also important to interpret their fate after ingestion and their effects on health, as anthocyanins may be consumed as a complex mixture of native forms, derivatives, and degradation products, which themselves can evolve in the digestive tract [3].

## 2. The Basis of Anthocyanin Chemistry

### 2.1. Anthocyanins Are Weak Diacids

**Figure 1:**
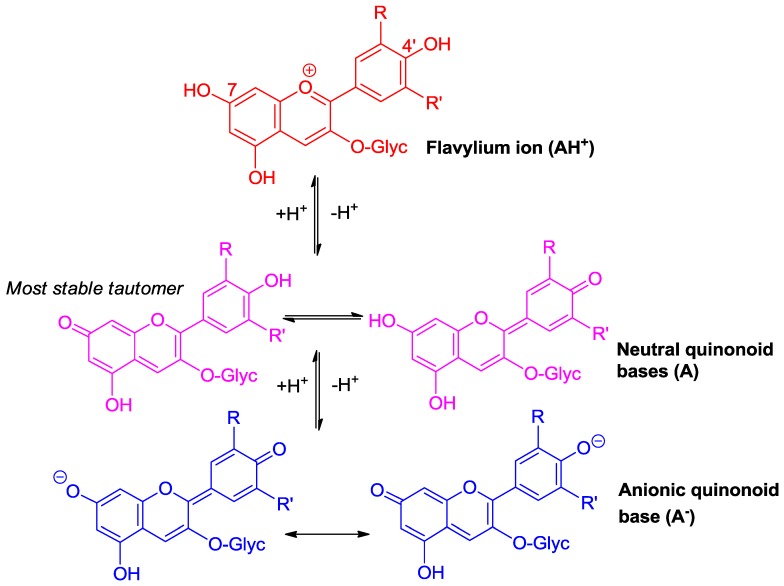
Flavylium ions are weak diacids.

**Figure 2:**
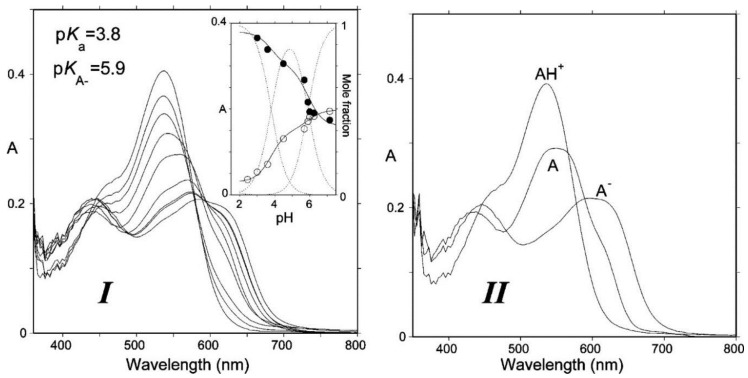
(**I**) Absorption spectra of Cat-Mv3Glc: pH jump from pH = 1.0 (100% flavylium) to pH 3.00, 3.59, 4.50, 5.70, 5.96, 6.25, and 7.15, respectively. Spectra recorded 10 ms after mixing (negligible water addition). (**II**) Spectra of the components obtained by mathematical decomposition. From [4] with permission of the *American Chemical Society*.

Due to conjugation with the electron-withdrawing pyrylium ring, the phenolic OH groups of the flavylium ion at C4′, C5, and C7 are fairly acidic [1,2]. In terms of structure–acidity relationships, it is clear that C7-OH is the most acidic group with a p*K*_a1_ of ca. 4, i.e., 6 p*K*_a_ units below the phenol itself. The corresponding neutral quinonoid base (Figure 1) can thus be considered to be the prevailing tautomer. At higher pH levels, a second proton loss from C4′-OH (p*K*_a2_ ≈ 7 for common anthocyanins) yields the anionic base with maximized electron delocalization over the three rings. Along this deprotonation sequence, the wavelength of maximal visible absorption typically shifts by 20–30 nm (AH^+^ → A), then by 50–60 nm (A → A^−^) (Figure 2), and the corresponding color turns from red to purple-blue [4].

### 2.2. Anthocyanins Are Hard and Soft Electrophiles

By analogy with enones, the C2 and C4 atoms of the pyrylium ring can be regarded as hard and soft electrophilic centers, respectively. Hence, they respectively react with hard (O-centered) and soft (S- and C-centered) nucleophiles, the first mechanism being driven by local charges and the second one by interactions between the frontier molecular orbitals (HOMO of nucleophiles and LUMO of electrophiles).

#### 2.2.1. Nucleophilic Addition at C2

**Figure 3:**
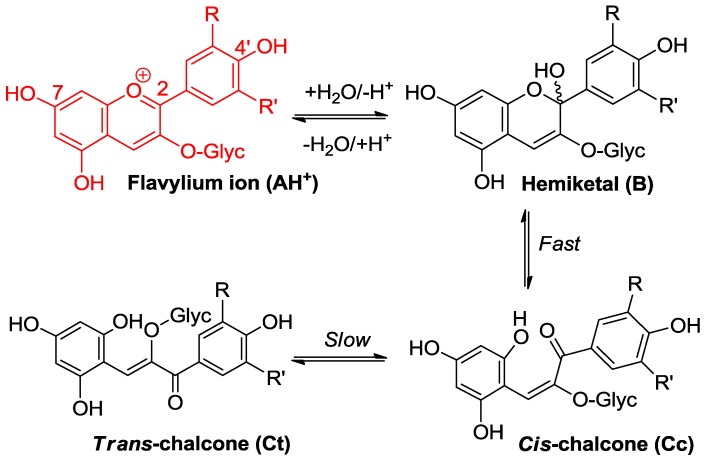
Flavylium ions are hard electrophiles reacting at C2 with O-centered nucleophiles, such as water (water addition followed by formation of minor concentrations of chalcones).

Water addition is the ubiquitous process taking place within aqueous anthocyanin solutions [1,2]. It leads to the colorless hemiketal (Figure 3) and can be characterized by the thermodynamic hydration constant *K*_h_, or as an acceptable approximation (chalcones making only a minor contribution, typically less than 20%, of the total pool of colorless forms), by the apparent constant *K*′_h_ connecting the flavylium ion and the colorless forms taken collectively. With common anthocyanins, p*K*′_h_ lies in the range of 2–3, which means that hydration is thermodynamically more favorable than proton transfer (p*K*′_h_ < p*K*_a1_). Fortunately, it is also much slower, and its pH-dependent kinetics can be quantified by the apparent rate constant of hydration (*k*_obs_) (Equation (1), *h* = [H^+^], *χ*_AH_ = mole fraction of AH^+^ within the mixture of colored forms [2,5]:

(1) $$k_{obs}=k_{h}\chi_{AH}+k_{-h}^{\prime }h=\frac{k_{h}}{1+K_{a1}/h+K_{a1}K_{a2}/h^{2}}+k_{-h}^{\prime }h.$$

*k*_h_ is the absolute rate constant of water addition, *k*′_−h_ is the apparent rate constant of water elimination (from the mixture of hemiketal and *cis*-chalcone in fast equilibrium), and *K*′_h_ ≈ *k*_h_/*k*′_−h_ (*trans*-chalcone neglected). Equation (1) can be easily understood by keeping in mind that the flavylium ion is the sole colored form that is electrophilic enough to directly react with water.

At a given pH, the initial visible absorbance (*A*_0_) (no colorless forms) and the final visible absorbance (*A*_f_) (hydration equilibrium established) can be easily related through Equation (2):

(2) $$\frac{A_{f}}{A_{0}}=\frac{1+K_{a1}/h+K_{a1}K_{a2}/h^{2}}{1+(K_{a1}+K_{h}^{\prime })/h+K_{a1}K_{a2}/h^{2}}.$$

Thus, the magnitude of color loss can be expressed as (Equation (3)):

(3) $$\frac{A_{0}-A_{f}}{A_{0}}=\frac{K_{h}^{\prime }/h}{1+(K_{a1}+K_{h}^{\prime })/h+K_{a1}K_{a2}/h^{2}}.$$

**Figure 4:**
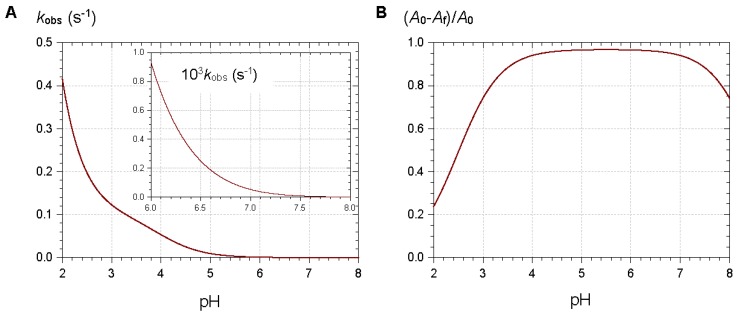
Simulations of the pH dependence of the apparent rate constant (**A**) and relative magnitude (**B**) of color loss. Selected values for parameters: p*K*_a1_ = 4, p*K*_a1_ = 7, p*K*′_h_ = 2.5, *k*_h_ = 0.1 s^−1^, *k*′_−h_ ≈ *k*_h_/*K*′_h_.

**Figure 5:**
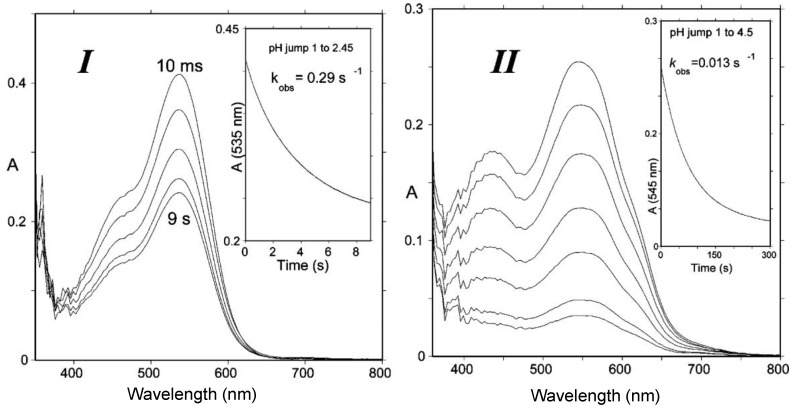
(**I**) Spectral changes of Cat-Mv3Glc between 10 ms and 9 s following a pH jump from pH = 1 to pH = 2.45; half-life of flavylium = 2.4 s. (**II**) pH jump from pH = 1 to pH = 4.5; half-life of quinonoid bases = 53.3 s. At pH = 6, the half-life of quinonoid bases ≈ 30 min. From reference [4] with permission of the *American Chemical Society*.

From typical values for the rate and thermodynamic constants of common anthocyanins, simulations of the pH dependence of the apparent rate constant and percentage of color loss can be constructed (Figure 4). The plots clearly show that the reversible color loss due to water addition to the flavylium ion becomes slower at higher pH (less flavylium in solution), whereas its magnitude becomes larger because of the higher stability of the colorless forms. The typical time-dependence of the visible spectrum during water addition is shown in Figure 5 [4].

Near neutrality water addition is so slow (fraction of flavylium ion < 0.1%) that the colored forms (mixtures of neutral and anionic bases) can, in principle, persist for hours. However, such a reasoning ignores the irreversible mechanisms of color loss taking place near neutrality as the anionic base is obviously much more sensitive to autoxidation (non-enzymatic oxidation by O_2_ triggered by transition metal traces) than the flavylium ion. These mechanisms will be addressed in Section 2.4.1.

#### 2.2.2. Nucleophilic Addition at C4

**Figure 6:**
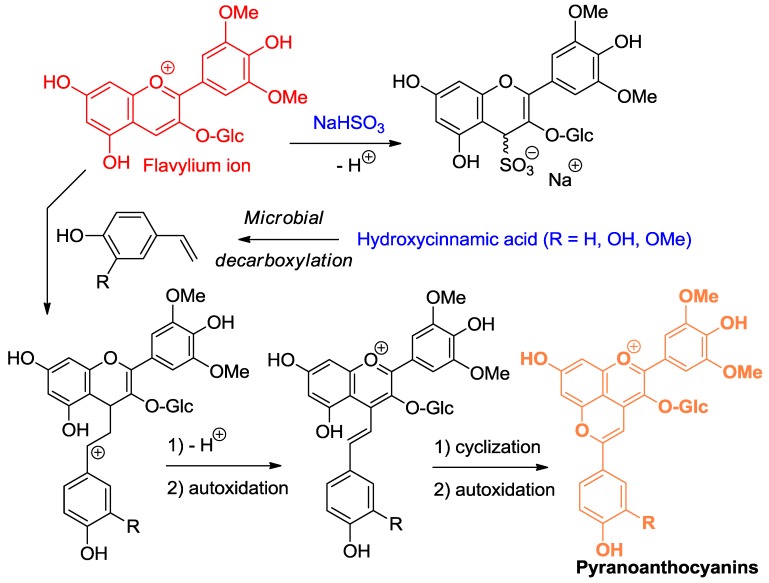
Flavylium ions are soft electrophiles that react at C4 with S- and C-centered nucleophiles, such as bisulfite and 4-vinylphenols.

Bisulfite is an antimicrobial and anti-browning agent that is frequently used in the food industry. As a S-centered nucleophile, it reversibly reacts with the flavylium ion at C4, thus yielding colorless adducts (Figure 6) [6]. No such adducts have been identified so far by simply reacting anthocyanins with natural thiols such as cysteine and glutathione (GSH). Unlike bisulfite, which is actually the conjugated base of SO_2_ (p*K*_a_ ≈ 1.8) and can coexist with the flavylium ion under acidic conditions, thiolate anions (p*K*_a_ = 8–9) are usually formed at much higher pH levels where the flavylium ion is only present as traces.

A variety of C-centered nucleophiles are also known to add to the flavylium ion, and this chemistry underlies the color changes observed in red wine upon aging [7]. In this context, the most important C-centered nucleophiles are electron-rich C–C double bonds, such as 4-vinylphenols (4-hydroxystyrenes), formed upon microbial decarboxylation of 4-hydroxycinnamic acids (Figure 6) and the enol forms of various aldehydes and ketones such as pyruvic acid and ethanal (acetaldehyde) [8,9]. In the process, new pigments, called pyranoanthocyanins, are formed, which are resistant to nucleophilic addition at C2 and C4 [10,11,12]. Their color (shifted to orange-red, compared to the corresponding flavylium ion) is thus more stable. Through their nucleophilic C6- and C8-atoms, flavanols and proanthocyanidins can also add to the electrophilic C4 center of anthocyanins [13]. However, the flavene intermediate thus formed is not accumulated and evolves through two possible routes: (a) under strongly acidic conditions (pH = 2), protonation at C3 allows a second nucleophilic attack by a nearby phenolic OH group of the tannin to yield a colorless product (see Section 2.3 for a similar mechanism); or (b) under moderately acidic conditions (pH = 3–6), dehydration with concomitant formation of an additional pyrane ring is favored and a new pigment bearing a xanthylium chromophore is formed.

With its enediol structure, ascorbate (vitamin C) can also react with flavylium ions at C4 but the corresponding adducts have not been reported so far.

### 2.3. Anthocyanin Hemiketals Are Nucleophiles

Basic organic chemistry teaches that electron-donating substituents of benzene rings accelerate aromatic electrophilic substitutions (*S*_E_*Ar*) and orient the entering electrophiles to the *ortho* and *para* positions. In that perspective, the phloroglucinol (1,3,5-trihydroxybenzene) ring (A-ring) of anthocyanins must be especially favorable to *S*_E_*Ar* as the three O-atoms combine their electronic effects to increase the reactivity of C6 and C8. However, the pyrylium ring (C-ring) of the flavylium ion (and, to a lesser degree, the enone moiety of chalcones) is strongly electron-withdrawing, so that only the hemiketal is expected to react by *S*_E_*Ar*.

**Figure 7:**
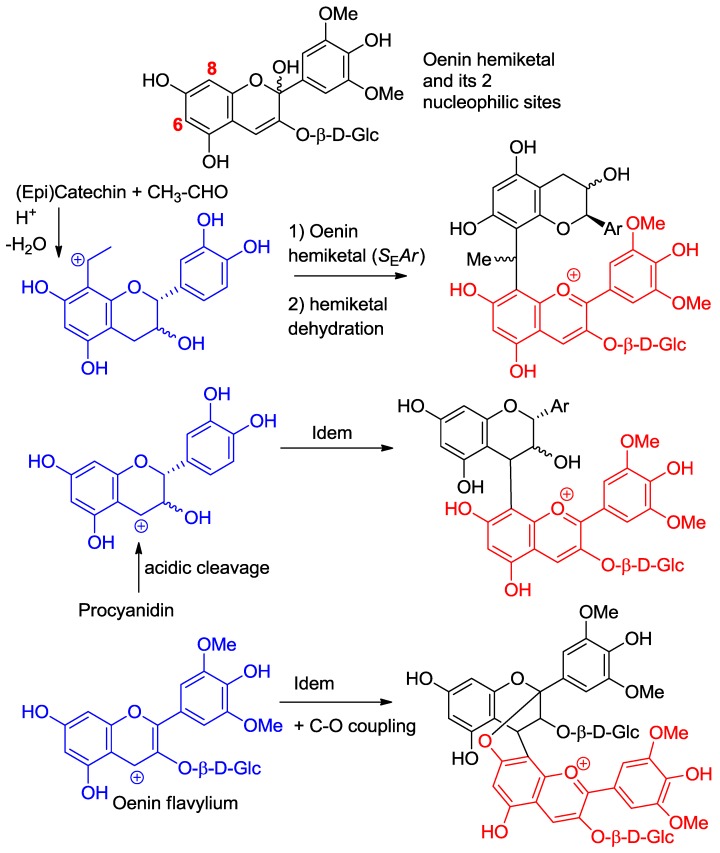
Anthocyanin hemiketals are nucleophiles reacting with carbocations (Ar = catechol ring).

Here, again, wine chemistry provides interesting examples of *S*_E_*Ar* between anthocyanins and various carbocations derived from other wine components (Figure 7) [7]. For instance, wine pigments in which anthocyanins and flavanols are linked though an ethylidene bridge between their C6- and/or C8-atoms are formed by double *S*_E_*Ar* between A-rings and ethanol [14,15]. The likely intermediates in the reaction are the 6- or 8-vinyl-flavanol and the 6- or 8-vinyl-anthocyanin hemiketals, the protonation of which delivers a benzylic cation that is directly involved in the *S*_E_*Ar* reaction. Of course, in addition to the cross reaction products, anthocyanin–ethylidene–anthocyanin and flavanol–ethylidene–flavanol adducts can also form oligomers and mixed oligomers [16]. Even, pyranoanthocyanins stemming from the nucleophilic attack of vinyl-phenols at C4 of anthocyanins can be produced.

Flavanol carbocations formed by acidic cleavage of the inter-flavan linkage of proanthocyanidins also react with anthocyanin hemiketals by *S*_E_*Ar* [17]. Interestingly, both direct and ethylidene-bridged flavanol–anthocyanin adducts are more purple than the native anthocyanin, but only the latter expresses a color that is stable, i.e., a flavylium nucleus that is less sensitive to water addition [4,18]. A possible explanation is that ethylidene-bridged flavanol–anthocyanin adducts are prone to non-covalent self-association by π-stacking, which provides a less aqueous environment for the flavylium nuclei.

Water elimination from the anomeric C-atom of the ellagitannin vescalagin (abundant in oak barrels) also delivers a carbocation for direct coupling with wine anthocyanins [19] and subsequent modest protection against water addition [20]. Finally, the anthocyanin hemiketal can react with the flavylium ion itself, and this pathway provides a route for anthocyanin oligomerization, a poorly documented mechanism as the corresponding oligomers are probably difficult to evidence and quantify. However, an oenin trimer has been found in Port wine, and its structure has been fully elucidated by NMR [21]. The two linkages are of the C4–C8 type. As in the direct flavanol–anthocyanin coupling (see Section 2.2.2), flavene intermediates evolve by C–O coupling and only the lower unit remains colored. Similar oligomers also occur with 3-deoxyanthocyanidins, e.g., in red sorghum, but the detailed structures remain unknown [22].

Anthocyanin hemiketals can also react by Michael addition with o-quinones formed by two-electron oxidation of catechols, such as epicatechin [13] and caffeoyltartaric acid [23].

### 2.4. Anthocyanins Are Electron-Donors

Many polyphenols, especially those containing electron-rich catechol (1,2-dihydroxybenzene) or pyrogallol (1,2,3-trihydroxybenzene) nuclei are good electron- or H-donors. Electron transfer is typically faster when the pH is raised, i.e., when the fraction of phenolate anion (a much better electron-donor than the parent phenol) increases. Electron transfer from phenols is involved in their oxidation mechanisms and also underlies the most common mechanism of antioxidant activity, i.e., the reduction of reactive oxygen species (ROS) involved in oxidative stress from plants to humans. Anthocyanins are known to be thermally unstable, especially under neutral conditions, and various degradation products have been identified. Their antioxidant activity has been also established in various chemical models. However, detailed knowledge on the mechanisms involved and on the relative contributions of the different colored and colorless forms is still missing.

#### 2.4.1. Oxidation

**Figure 8:**
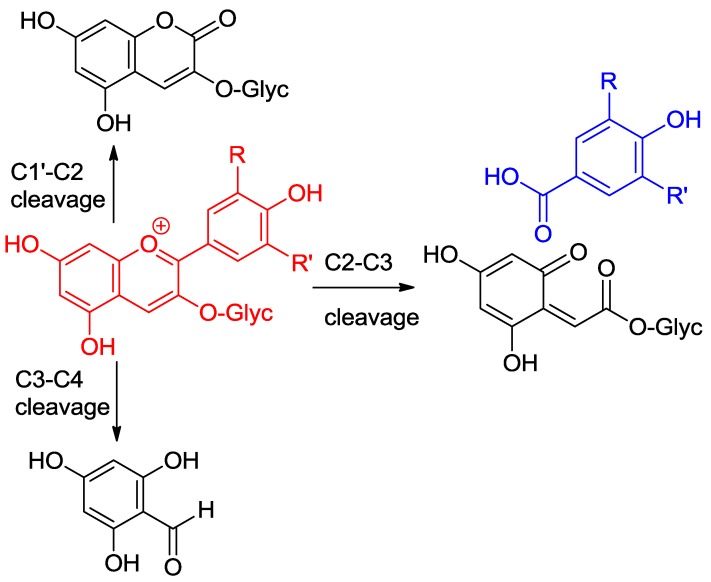
Pathways of anthocyanin degradation.

**Figure 9:**
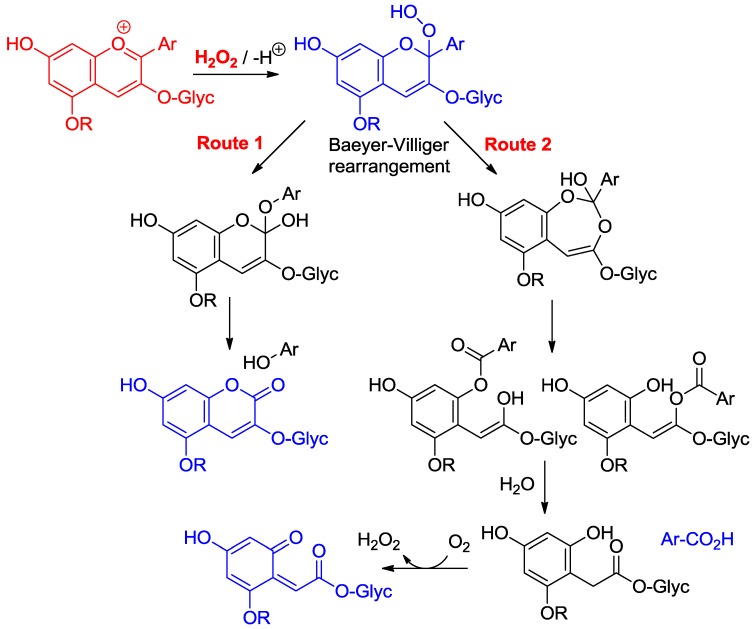
Possible mechanisms of anthocyanin degradation with pre-formed hydrogen peroxide.

**Figure 10:**
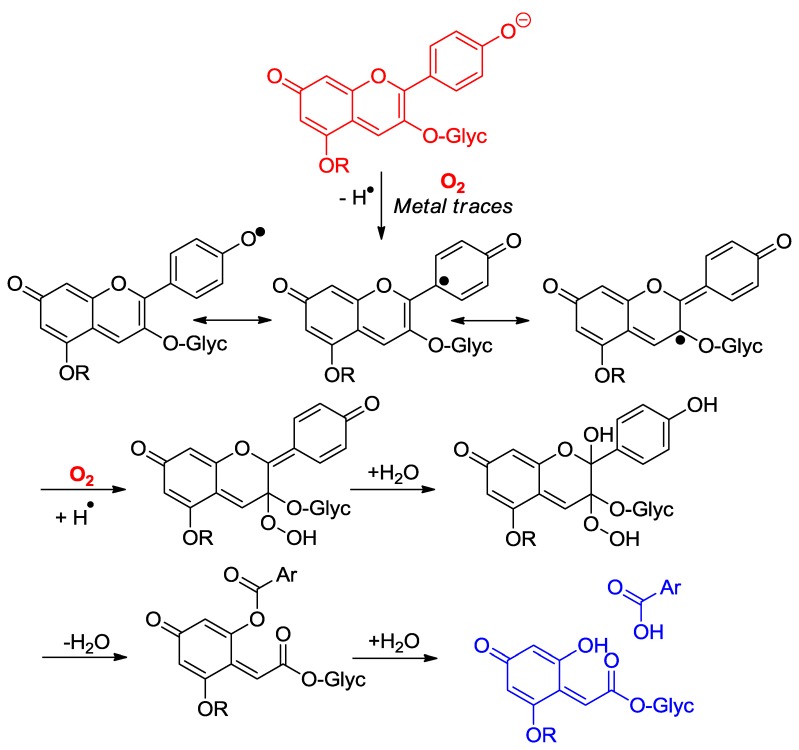
Possible mechanisms of anthocyanin degradation without pre-formed hydrogen peroxide.

Anthocyanins are among the least thermally stable flavonoids. Anthocyanidins, the corresponding aglycones, are actually only stable under highly acidic conditions and are extensively degraded in less than one hour under physiological conditions (pH = 7.4, 37 °C) [24,25]. From the structure of the degradation products, it is clear that a combination of hydrolytic and autoxidative pathways operate, leading to cleavage of the C2–C1′, C2–C3 and C3–C4 bonds (Figure 8) [13,26,27]. A mechanism involving pre-formed hydrogen peroxide actually accounts for the formation of some cleavage products (Figure 9). The critical step is the addition of H_2_O_2_ (a hard nucleophile) at C2 of the flavylium ion, followed by Baeyer–Villiger rearrangement, which opens routes for cleavage of the C2–C1′ and C2–C3 bonds [13,26]. However, the preliminary formation of H_2_O_2_ remains unclear and must involve the direct autoxidation of anthocyanins. Thus, an alternative mechanism beginning by electron or H-atom transfer (mediated by unidentified transition metal traces) from the anionic or neutral base to O_2_ would deliver a highly delocalized radical that is susceptible to O_2_ addition at different centers (Figure 10). The cleavage of hydroperoxide intermediates thus formed could also yield the degradation products detected.

#### 2.4.2. Antioxidant Activity

Anthocyanins under their native forms can transfer electrons to ROS and could, therefore, provide protection to important oxidizable biomolecules, such as polyunsaturated fatty acids (PUFAs), proteins, and DNA. The relevance of such phenomena is probably much higher in food preservation than in nutrition and health, given the current knowledge on anthocyanin bioavailability (see Section 3). In this section, we simply mention that anthocyanins can indeed effectively reduce one-electron oxidants such as the stable radical DPPH (2,2-diphenyl-1-picrylhydrazyl). Structure–activity relationships show that hydroxylation at C3′ and C5′ increases the H-donating capacity, thus suggesting that the B-ring is primarily involved in electron donation [28]. Comparing oenin and the flavanol catechin shows that the transfer of the first (most labile) H-atom to DPPH is roughly as fast for both flavonoids but that oenin reduces at least twice as many radicals than catechin (Table 1) [29]. This advantage must be rooted in the extensive oxidative degradation undergone by oenin during the DPPH-scavenging process with the transient formation of intermediates (possibly, syringic acid) retaining a substantial electron-donating activity. It is also remarkable that the wine pigments combining the oenin and catechin units retain a high but contrasting DPPH-scavenging activity [29]: the direct coupling between the two flavonoid units results in a faster first H-atom transfer (higher *k*_1_) but markedly lowers the total number of radicals reduced (*n*_tot_), whereas the coupling through an ethylidene bridge apparently leaves each unit free to independently react with DPPH (*k*_1_ almost unchanged, approximate additivity in the *n*_tot_ value), as observed with the equimolar oenin–catechin mixture (Table 1).

**Figure 11:**
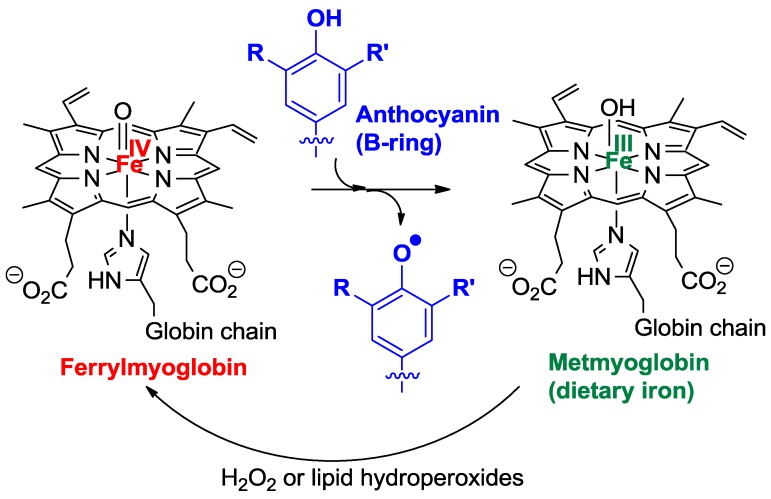
Possible mechanisms for the antioxidant activity of anthocyanins in food and in the gastro-intestinal tract.

Oenin, catechin, and wine pigments were also compared for their ability to inhibit the peroxidation of linoleic acid induced by dietary heme iron in acidic micelle solutions, a chemical model of postprandial oxidative stress in the stomach [29]. As hydrophilic antioxidants, polyphenols are known to act at the initiation stage by reducing the hypervalent iron species (Fe^IV^) involved in the generation of propagating lipid peroxyl radicals (Figure 11) [30] which, on the other hand, are directly reduced by the typical chain-breaking amphiphilic antioxidant α-tocopherol (vitamin E). The highly hydrophilic oenin was found to be less potent than catechin in the inhibition, but coupling both flavonoids via an ethylidene bridge improves their efficiency (Table 1).

Acylation by electron-rich hydroxycinnamic acids, such as sinapic and ferulic acids, potentiates the capacity of anthocyanins to inhibit the diazo-initiated autoxidation of styrene in acetonitrile. In particular, a higher rate constant and stoichiometric factor of radical scavenging were obtained for acylated (*vs*. non-acylated) anthocyanins [31]. Curiously, this trend could not be confirmed for the peroxidation of linoleic acid in micelles, as if the intrinsic differences in electron-donating activity were counterbalanced by differences in the partition of anthocyanins between micelles and the aqueous phase.

### 2.5. Anthocyanin Complexes

Phenolic nuclei have an intrinsic ability to develop molecular (non-covalent) interactions as they combine flat polarizable apolar surfaces (the aromatic nuclei) for strong dispersion interactions and polar OH groups that are susceptible to acting as H-bond donors and acceptors.

#### 2.5.1. Self-Association and Co-Pigmentation

One of the most remarkable properties of the anthocyanin chromophores is their ability to develop π-stacking interactions [32,33,34], mostly driven by dispersion interactions and the concomitant favorable release of water molecules from the solvation shells of the interacting nuclei, known as the hydrophobic effect. Owing to their planar structures and extended electron delocalization over the three rings, the colored forms are much more prone to π-stacking interactions than the colorless forms, for which such interactions, although not necessarily absent, are typically neglected. Examples of π-stacking interactions with anthocyanins are self-association and binding between anthocyanins and other phenols, a phenomenon called co-pigmentation. The affinity of co-pigments for a given anthocyanin (as measured by the corresponding thermodynamic binding constant) decays along the series: planar flavonoids (flavones, flavonols) > non-planar flavonoids (catechins), hydroxycinnamic acids > hydroxybenzoic acids [32]. As for self-association, it is stronger for the neutral base than for the flavylium ion and the anionic base, as the latter stacks are destabilized by charge repulsion.

**Figure 12:**
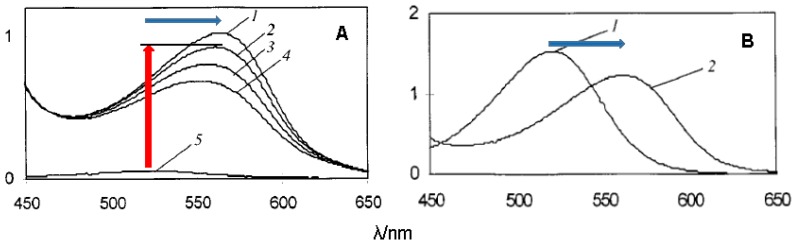
Co-pigmentation of malvin (malvidin 3,5-diglucoside, 50 µM) by rutin bis(hydrogensuccinate) (mixture of 3 regioisomers, 200 equiv.). (**A**) pH = 3.5, malvin + co-pigment at T = 15.5 (1), 25.0 (2), 35.0 (3), 44.2 (4) °C, malvin alone at T = 25.3 °C (5). (**B**) pH = 0.9, T = 25.0 °C, malvin alone (1), malvin + co-pigment (2). Adapted from reference [35].

The spectral consequences of co-pigmentation are summarized in Figure 12 with malvin (malvidin 3,5-di-*O*-β-glucoside) and a highly water-soluble rutin (quercetin 3-*O*-β-rutinoside) derivative [35]. In strongly acidic solutions (negligible water-to-flavylium addition), π-stacking interactions between the two partners promote bathochromism as a consequence of co-pigment-to-pigment charge transfer. Changes in color intensity simply reflect differences between the molar absorption coefficients of free and bound pigments. Under the mildly acidic conditions typically encountered in natural media, pigment–co-pigment interactions also promote hyperchromism, which can be understood as a shift in the now established flavylium–hemiketal equilibrium toward the colored form, which is selectively stabilized by its association with the co-pigment. This combination of bathochromic and hyperchromic shifts makes co-pigmentation one of the most important mechanisms for color variation and stabilization in plants. It can also be noted that heating usually attenuates the hyperchromic shift (Figure 12) as a consequence of the exothermic character of co-pigmentation (∆*H*^0^ < 0).

**Figure 13:**
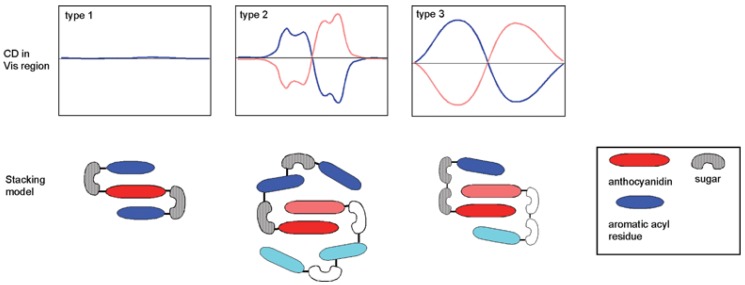
Acylated anthocyanins: discrimination of intramolecular co-pigmentation (type 1) and self-association (types 2 and 3) by circular dichroism (pink or blue CD spectra depending on the chirality of the stacks). From [34] with permission of the *Royal Society of Chemistry*.

The possibility of developing π-stacking interactions increases with the acylation of anthocyanins on their glycosyl moieties by hydroxycinnamic acid (HCA) residues. Indeed, depending on the location and number of HCA residues, different spatial arrangements can be observed (Figure 13) [34]:

- Intramolecular co-pigmentation: π-stacking interactions promote a conformational folding of the pigment bringing one or more HCA residue(s) into contact with the chromophore;
- Enhanced self-association: the HCA residues can stabilize the chiral stacking of chromophores evidenced by circular dichroism.

**Figure 14:**
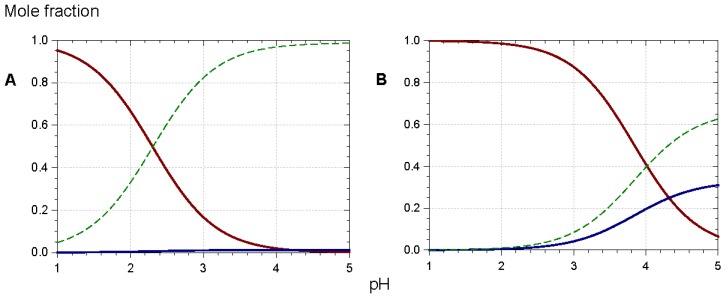

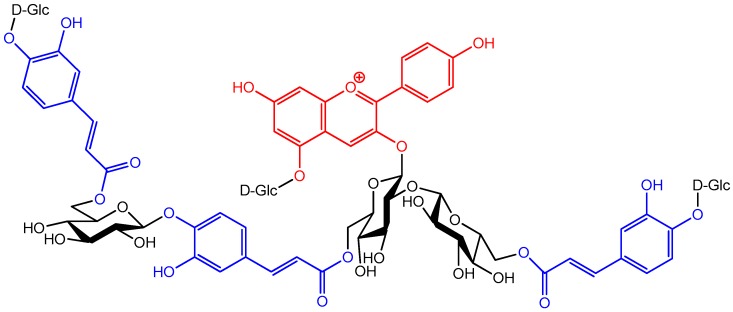
Triacylated (**B**) vs. non-acylated (**A**) *Morning glory* (*Pharbitis nil*) anthocyanins: equilibrium distribution of anthocyanin species in aqueous solution. Red solid line: flavylium ion, blue solid line: neutral base, dotted green line: total colorless forms. Parameters for plots are p*K*’_h_ = 2.30, p*K*_a1_ = 4.21 (**A**); p*K*’_h_ = 4.01, p*K*_a1_ = 4.32 (**B**). From [36,37].

In such assemblies, the flavylium nucleus has restricted access to the water solvent. Consequently, the thermodynamics of water addition are less favorable (increased p*K*′_h_), and the percentage of colored forms at equilibrium increases [5,36,37,38]. For instance, at pH = 3, ca. 90% of the triacylated *Morning glory* pigment is still in colored form (mostly flavylium) vs. 15% for its non-acylated counterpart (Figure 14). Its vulnerability to water addition prevents the non-acylated pigment from accumulating the neutral quinonoid base at higher pH levels, and the corresponding solutions are almost colorless. In contrast, 30% of the triacylated pigment is present as the colored neutral base at pH = 5. Moreover, the π-stacking interactions developed by the triacylated flavylium ion induce a 20 nm bathochromic shift of its λ_max_ compared to its non-acylated counterpart.

**Figure 15:**
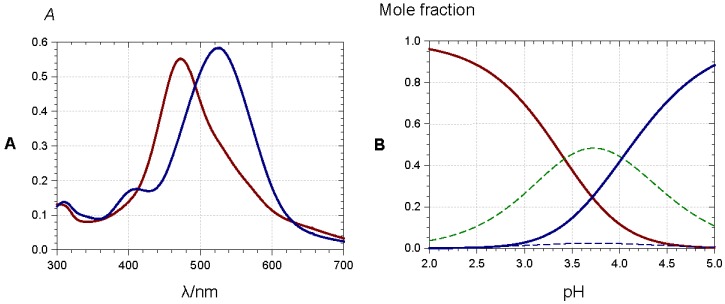
(**A**) 3′,4′-Dihydroxy-7-*O*-β-d-glucopyranosyloxyflavylium (50 µM) in a pH 4 buffer (0.1 M acetate), red spectrum: before hydration, blue spectrum: 10 min after addition of Al^3+^ (4 equiv.); (**B**) equilibrium distribution of species in aqueous solution. Red solid line: flavylium ion, blue dotted line: neutral base, dotted green line: total colorless forms, blue solid line: Al^3+^ complex. Parameters for plots are p*K*’_h_ = 3.42, p*K*_a1_ = 4.72, *K*_M_ = 2 × 10^−4^. From [39].

Anthocyanins with an *o*-dihydroxy substitution on their B-ring (cyanidin, delphinidin, and petunidin derivatives) also bind hard metal ions, such as Al^3+^ and Fe^3+^, in mildly acidic to neutral solution. As the anthocyanin binds as the quinonoid base with additional proton loss from C3′-OH, bathochromism is observed with additional ligand-to-metal charge transfer with Fe^3+^ (Figure 15).

At least in mildly acidic solution, metal binding is restricted to the colored forms and thus efficiently competes with the hydration equilibrium, thereby preventing the formation of the colorless forms. In the most sophisticated assemblies, metal binding and π-stacking interactions combine, thus providing the most common mechanism towards the formation of stable blue colors [34,40,41]. In the so-called metalloanthocyanins, a fixed metal–pigment–co-pigment stoichiometry of 2:6:6 is observed: three anthocyanins bind to each metal ion and two equivalent complexes assemble by left-handed π-stacking interactions between the chromophores. Then, three pairs of flavone or flavonol co-pigments in left-handed π-stacking intercalate between the pairs of stacked anthocyanins. In this intercalation, right-handed pigment–copigment π-stacking occurs. Large-scale aggregation of acylated anthocyanins can also result in the formation of highly colored assemblies within the vacuole (the so-called anthocyanin vacuolar inclusions) [42], the organelle where anthocyanins are stored in plant cells.

#### 2.5.2. Binding to Biopolymers

Despite the potential significance of such associations in food chemistry and nutrition, the ability of anthocyanins to bind proteins and polysaccharides is still poorly documented at the molecular level. This paragraph focuses on anthocyanins (glycosides), although anthocyanidins are also commonly investigated. Indeed, aglycones are chemically unstable in mildly acidic and neutral conditions and may be substantially degraded over the duration of analysis.

Saturation transfer difference (STD)-NMR was used to probe the binding of cyanidin and delphinidin 3-glucosides to pectin from citrus fruits (MM = 111 kDa) [43]. Indeed, magnetization transfer (requiring proton pairs distant by less than 0.5 nm) from irradiated pectin protons to anthocyanin protons provided direct evidence that the two partners are in close contact. STD titrations at pH = 4.0 and pH = 1.5 suggest that the flavylium ion has a higher affinity for pectin than the hemiketal. Assuming the Scatchard model (*n* identical binding sites having the same binding constant, *K*_b_), pectin was found to bind 180–600 anthocyanin units depending on the selected anthocyanin and pH. The corresponding *K*_b_ values are very weak (<10^3^ M^−1^). Thus, the picture emerging from this study is that anthocyanins (as individual species or non-covalent oligomers) provide a coating of the pectin’s surface through the development of very weak interactions (van der Waals contacts, H-bonds).

The quenching of intrinsic protein fluorescence by increasing ligand concentrations is a classical method to probe ligand–protein binding and to extract binding parameters. As anthocyanins typically absorb light at the protein’s excitation and emission wavelengths, corrections for these inner-filter effects should be applied [44], which are not systematic [45] and thus lead to discrepancies in *K*_b_ values as well as in enthalpy and entropy changes. With human serum albumin (HSA), a globular protein, 1:1 binding is observed with a *K*_b_ in the order of 10^5^ M^−1^ [44,45], meaning a moderate affinity. The influence of the pH (from pH = 4 to pH = 7.4) on the binding strength is very modest [44]. Competition with probes of a known binding site (ibuprofen, warfarin) enables location of the anthocyanin binding site, a hydrophobic pocket lined by positively charged amino-acid residues (Arg, Lys) for possible accommodation of the anionic base [45]. As for the weakly structured salivary proteins, interaction with malvidin-3-glucoside (probed by STD-NMR) was found to be much weaker (*K*_b_ ≈ 500 M^−1^) and largely pH-independent (same affinity at pH = 1.0 and pH = 3.4), which suggests that the hemiketal and flavylium ions bind with close affinities [46]. Electrospray ionization MS revealed the formation of soluble aggregates involving 2–6 anthocyanin units and 1–4 peptides (proline-rich proteins or histatin). STD-NMR was also used to investigate the binding of keracyanin (cyanidin 3-rutinoside) to wheat flour gliadins at pH = 2.5 [47]. Protons C2′-H, C5′-H, C6-H and C8-H appear to be primarily involved in the binding. At this low pH, the corresponding aglycone (cyanidin) is stable and can be also investigated. Its affinity for gliadins appears higher based on the strong shielding of its proton signals when gliadins are added (confirmed by the large retention of cyanidin in the centrifugation pellet: up to 80% vs. only 8% for keracyanin). However, STP-NMR did not point protons specifically involved in the interaction. Cyanidin 3-glucoside expresses a rather high affinity for sodium caseinate (NaCas) [48]. Two binding sites were identified at pH = 2 and pH = 7, one of high affinity (*K*_b_ ≈ 1–7 × 10^6^ M^−1^ depending on pH and T) and a second of lower affinity (*K*_b_ ≈ 2–7 × 10^5^ M^−1^). For both sites, the binding was found to be exothermic at pH = 7 but endothermic at pH = 2 and thus is driven by a favorable entropy, which could point to a large contribution of the hydrophobic effect. Interestingly, NaCl addition gradually cancels cyanidin 3-glucoside–NaCas binding at pH = 7 but has no effect at pH = 2. In contrast to the high affinity of cyanidin 3-glucoside for NaCas, malvidin 3-glucoside only weakly binds to α- and β-caseins [49] and to β-lactoglobulin [50] (1:1 binding with *K*_b_ < 10^3^ M^−1^).

Unlike co-pigmentation, the binding of anthocyanins to biopolymers does not trigger spectacular spectral changes. For instance, in the presence of various polysaccharides [51], no change in the wavelength of maximal visible absorption (λ_max_) was observed. Interactions of anthocyanins with cellulose, oat bran, and lignin is associated with a weak hypochromic effect, whereas an opposite effect (weak hyperchromism) is observed with highly methylated apple pectins. Sugar beet pectins have been shown to promote strong bathochromism in solutions of blackcurrant anthocyanins (cyanidin and delphinidin glycosides), but this effect is due to endogenous iron ions (bound to the polysaccharide) forming blue chelates with the pigments [52]. In agreement with the small spectral changes observed, the binding of anthocyanins to pectin does not significantly affect the thermodynamic constants of the acid–base and hydration equilibria [43]. In other words, all anthocyanin forms (colored or colorless) bind pectin with close affinities. This apparent discrepancy with the STD-NMR data (stronger flavylium–pectin binding) might be due to anthocyanin self-association, which probably is significant in the concentrated solutions used in the STD-NMR experiments. In contrast, the flavylium cation of the pyranoanthocyanin portisin is strongly stabilized by interactions with anionic wood lignosulfates as evidenced by its much weaker acidity in the presence of the polysaccharide (p*K*_a1_ = 6.6 vs. 4.6 for portisin alone) [53].

### 2.6. Anthocyanins in the Excited State

**Figure 16:**
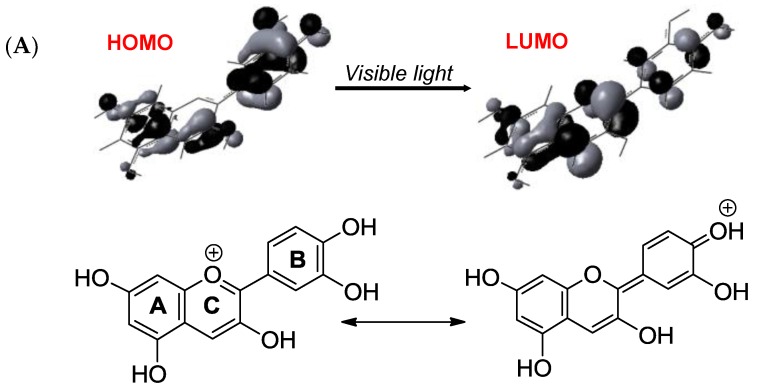

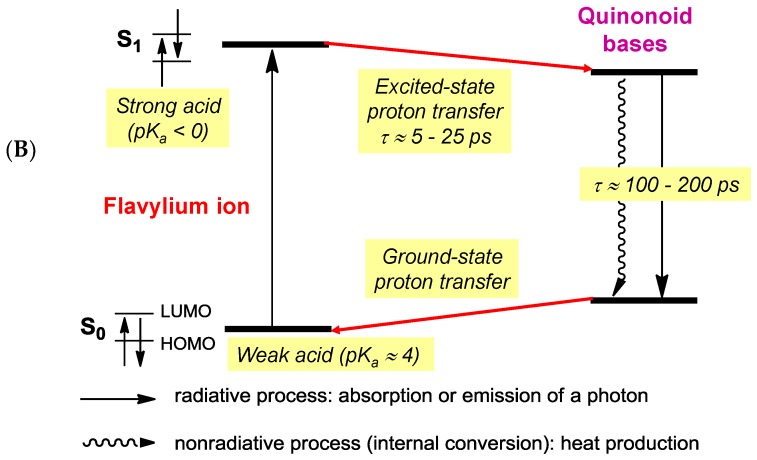
(**A**) Frontier MOs of the flavylium ion of cyanidin (from reference [55]) and its most representative mesomeric forms in the ground state (left) and first excited state (right). (**B**) The fate of free anthocyanins in the excited state (from references [54,56]).

**Figure 17:**
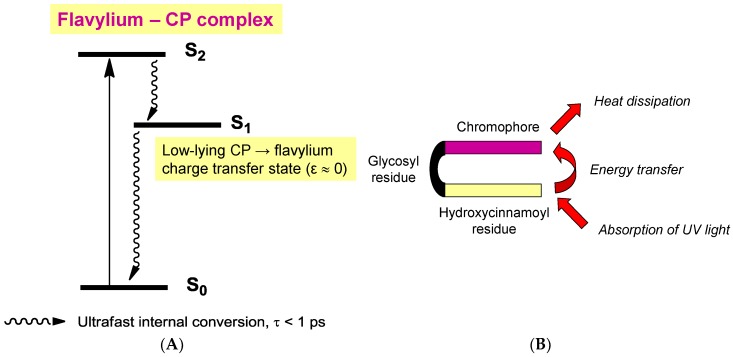
The influence of co-pigmentation on the fate of anthocyanins in the excited state. (**A**) Intermolecular co-pigmentation (from reference [57]). (**B**) Intramolecular co-pigmentation (from reference [58]).

Although their main function is to absorb visible light and express color, anthocyanins are intrinsically poorly fluorescent with quantum yields typically lower than 4 × 10^−3^ (meaning that less than one photon out of 250 absorbed is actually re-emitted) [54]. Indeed, the fate of anthocyanins after absorption, i.e., once in the excited state, is a difficult question that must be addressed by sophisticated fast techniques, such as time-resolved fluorescence and transient absorption-emission spectroscopies. In the HOMO → LUMO transition accompanying the absorption of visible light by the flavylium ion, electron transfer from the B-ring to the A-/C-rings takes place (Figure 16) [55]. In the excited state, the flavylium ion is a strong acid (p*K*_a_ < 0) that transfers a proton to the solvent on a picosecond timescale (20 ps for pelargonin at pH = 1) [54,56]. In the next step, the quinonoid base in the excited state is deactivated by a combination of radiative (fluorescence) and non-radiative (heat) processes and then captures a proton in the ground state to form the ground state flavylium ion. In other words, the quinonoid base is responsible for the (weak) fluorescence observed for anthocyanins even in strongly acidic solution. In the presence of a co-pigment, other mechanisms (Figure 17) supersede the fast flavylium deprotonation observed with free anthocyanins [57] in the following ways: (a) within the complex in the excited state, through ultrafast internal conversion (<1 ps) via a low-energy co-pigment-to-pigment charge transfer state, resulting in static fluorescence quenching; and (b) for the fraction of free anthocyanin, diffusion-controlled electron transfer from the co-pigment to the flavylium ion in the excited state, resulting in dynamic fluorescence quenching. The mechanism of energy dissipation by ultrafast internal conversion has been confirmed for the folded conformation of a cyanidin glycoside acylated by *p*-coumaric acid [58]. In addition, fast energy transfer to the chromophore following absorption of UV light by the acyl residue operates (Figure 17), thereby conferring acylated anthocyanins to have an important role in plant photoprotection. 

## 3. The Importance of Anthocyanin Chemistry in Food and Nutrition

### 3.1. Formulation of Anthocyanins for Food Applications

Anthocyanin degradation typically occurs during thermal processing and storage. The knowledge on anthocyanin–biopolymer interactions can be applied to devise formulations for improved chemical stability. Degradation studies aimed at demonstrating the protection afforded by biopolymers may be limited to monitoring the color loss under given conditions of pH, temperature, and light exposure. More information is obtained when samples are also acidified to pH 1–2 for quantification of the residual flavylium ions by HPLC or by UV-visible spectroscopy. With this approach, color loss (directly observed at the monitoring pH), which combines the reversible water addition and irreversible phenomena (hydrolysis, autoxidation), and true anthocyanin loss (irreversible component), can be distinguished.

In the simplest experiments involving modeling beverages, solutions of anthocyanins and soluble biopolymers are heated, and their color or residual anthocyanin concentration is monitored as a function of time. For instance, yeast mannoproteins (0.5% *w*/*w* for both anthocyanins and mannoproteins) increase the half-life of color loss by a factor of 5.4 in experiments conducted at pH = 7 and T = 80 °C or 126 °C (modeling pasteurization or sterilization) [59]. Similarly, the color loss in solutions of purple carrot anthocyanins at pH = 3.0 and T = 40 °C (in light) was shown to be inhibited by the addition of gum arabic (0.05–5.0%) with maximal stability observed at 1.5% (50% color retention after 5 days, vs. 20% in control) [60]. Similar observations were made with pectins or whey proteins (1%), the best result being obtained with heat-denatured whey proteins (70% color retention after 7 days at 40 °C, vs. 20% in control) [61]. In these works, fluorescence quenching experiments suggest that color protection involves direct interactions between anthocyanins and proteins (including the glycoprotein of gum arabic). However, the mechanism of protection remains largely unknown. It may be speculated that biopolymers mostly act by providing a more hydrophobic environment to anthocyanins, resulting in slower hydrolysis (despite the weak impact on the hydration equilibrium itself, see Section 2.5.2) and/or by scavenging transition metal traces acting as initiators/catalysts of anthocyanin autoxidation.

A more sophisticated approach consists of preparing solid micro- or nanoparticles as delivery systems for anthocyanins. For instance, nanoparticles of whey proteins and beet pectin can be loaded with anthocyanin extracts with a higher efficiency (55%) when anthocyanins are added prior nanoparticle formation [62]. However, when dispersed in pH 4 solution, these nanoparticles do not show improved color stability. Particles of chitosan and carboxymethylchitosan (CMC) loaded with anthocyanins (size ≈ 200 nm, encapsulation efficiencies ranging from 16 to 44% depending on the CMC/chitosan proportions) can be simply prepared by mixing at pH = 5–6 followed by centrifugation [63]. The thermal stability of encapsulated anthocyanins was shown to greatly improve: 12% degradation after 3 days at 40 °C, vs. 90% in the control (no particles). Similar protection was observed in samples exposed to white light for 10 days (−20% vs. −80%). Sulfonylated polysaccharides, such as dextran sulfate and carrageenans, can also be used to encapsulate bilberry anthocyanins from acidic solutions (pH ≈ 3) with high efficiency and improved stability [64,65]. The binding of isotherms and HPLC analysis showed that the binding is selective of anthocyanins (the other phenols remaining in solution) and is stronger when the sulfonylation degree is higher. These data strongly suggest that the encapsulation is driven by ionic flavylium–sulfate interactions. Interestingly, the nanoparticles are gradually dissociated under near neutral conditions modeling the small intestine, which is desirable for subsequent intestinal absorption. Combining chitosan and cellulose nanocrystals at pH 2–3 also allows the formation of nanoparticles with high affinity for anthocyanins (up to 94% encapsulation) [66]. When cellulose is replaced by sodium tripolyphosphate, a reticulating agent for the polycationic chitosan chain, gel microcapsules (size ≈ 34 µm, encapsulation yield ≈ 33%) are formed. Finally, large hydrogel particles (size ≈ 2–3 mm) combining alginate and pectin can be used for encapsulation of anthocyanin-rich extracts under acidic conditions (pH = 1–3), and they are released upon dissolution at higher pH [67]. When exposed to white light, the half-life values of anthocyanins in hydrogel, hydrogel particles dispersed in pH 3 solution, and in a control solution (pH = 3) were 630 h, 277 h, and 58 h, respectively.

Interestingly, anthocyanin-rich blackcurrant extracts can be incorporated into bread [68]. Replacing wheat flour by a mixture of gluten and starch led to markedly decreased anthocyanin concentrations (especially, for delphinidin glycosides, which are most sensitive to oxidation). This suggests that other flour proteins (e.g., albumins, globulins) and non-starch polysaccharides (e.g., hemicelluloses, β-glucans) may be important to provide chemical stability to anthocyanins in such matrices.

### 3.2. The Fate of Anthocyanins in Humans, Consequences on the Possible Effects on Health

The bioavailability of phenolic compounds has been largely elucidated over the last decades [69]. This knowledge, which is crucial to the interpretation of the possible effects on health, encompasses the bioaccessibility (the release of phenols from the food matrix during digestion), intestinal absorption, metabolism, transport, distribution to tissues, and excretion of dietary phenols and their metabolites. Anthocyanins have emerged as poorly bioavailable micronutrients as judged from the low concentrations (generally, <0.1 µM) of native forms (mostly, anthocyanidin glucosides) and anthocyanidin conjugates detected in the general blood circulation [70,71]. These derivatives are formed in the small intestine after enzymatic hydrolysis by membrane-bound lactate phlorizin hydrolase or by cytosolic β-glucosidase, and subsequent conjugation by *O*-glucuronidation, *O*-methylation, and/or *O*-sulfonylation. The detection of native forms in the blood circulation is not equivalent to other flavonoid glucosides and could be due to partial absorption from the stomach. This early absorption has been demonstrated in cell and animal models [72,73,74] and has been proposed to involve the organic anion transporter bilitranslocase in the gastric epithelium [72].

Most importantly, recent investigations, in particular using ^13^C-labelled compounds [3], have shown that the bulk of the ingested amount of anthocyanins is actually converted into simple phenolic compounds (Table 2), as a consequence of (a) the chemical instability (under near neutral conditions) of anthocyanins and, especially, of anthocyanidins [24] and (b) the extensive catabolism by the colonic microbiota of the fraction reaching the large intestine. These simple metabolites, which themselves can be further conjugated by intestinal and hepatic enzymes, have been found in the blood circulation in much higher concentration than anthocyanidin derivatives [3,75].

In agreement with the strong in vivo catabolism of anthocyanins, in vitro digestion models have shown that whereas anthocyanins are readily released into the acidic gastric compartment and relatively stable, they undergo substantial degradation in the near neutral upper intestinal compartment, possibly because of autoxidation [76,77]. However, this chemical instability could be overestimated in in vitro models, as the O_2_ content is higher than under real physiological conditions. As a striking example, protocatechuic acid (PCA, 3,4-dihydroxybenzoic acid), recovered in blood and fecal samples, was shown to represent more than 70% of the ingested dose of the cyanidin *O*-glucosides from blood orange juice [75]. Interestingly, PCA can be formed by chemical oxidative degradation of anthocyanins and anthocyanidins (Figure 8, Figure 9 and Figure 10). However, it must be noted that anthocyanins bearing an electron-rich B-ring (e.g., cyanidin and delphinidin glycosides) must be much more prone to oxidative degradation than, for instance, pelargonidin derivatives [78], which indeed could be detected in higher concentrations (0.2–0.3 µM) in the blood [79].

In the digestive tract, anthocyanins may also modulate the digestion and uptake of nutrients by interacting with intestinal α-glucosidase [80]. They could, as well, attenuate oxidative stress in the digestive tract, for instance, by inhibiting the peroxidation of dietary lipids induced by heme iron [29,81]. After intestinal absorption, anthocyanin derivatives are probably transported in the blood in moderate association with serum albumin [45] before distribution to tissues, which, again, could involve bilitranslocase, as evidenced in the kidneys of rats [82].

Most importantly, it must be kept in mind that the degradation products of anthocyanins, which are formed in the digestive tract and are generally much more abundant than the residual anthocyanidin derivatives, could mediate most of the potential health effects of anthocyanins [83,84], which remains intriguing given their chemical simplicity [3] (Table 2). However, redox-active compounds, such as PCA, could indeed participate in regulating the expression of genes associated with transcription factors susceptible to redox activation. Such mechanisms could, at least partly, underline the induction of antioxidant defense via the Nrf2 pathway and the reduction of inflammation via NF-κB inhibition observed in cells and in rodents with cyanidin derivatives [85] or PCA itself [86].

## Figures and Tables

**Table 1:** Antioxidant activity of malvidin 3-*O*-β-d-glucoside (oenin) and related pigments: reduction of the DPPH (2,2-diphenyl-1-picrylhydrazyl) radical (MeOH, 25 °C, ^1^ and ^2^) and inhibition of heme-induced peroxidation of linoleic acid (0.1 mM linoleic acid in acetate buffer + 2 mM Tween-20, 0.1 µM metmyoglobin, pH = 4, 37 °C, ^3^). From reference [29].

Antioxidant	*n*_tot_^1^	*k*_1_/s^−1 2^	IC_50_/μM ^3^
Oenin	11.26 (±0.08)	910 (±70)	0.68
Catechin	4.86 (±0.03)	1200 (±110)	0.27
Oenin + Catechin (1:1)	14.04 (±0.10)	1160 (±330)	nd
(*R*)-Catechin-8-CHMe-8-Oenin	14.56 (±0.03)	1000 (±320)	0.15
(*S*)-Catechin-8-CHMe-8-Oenin	14.61 (±0.18)	600 (±120)	0.41
Catechin-4,8-Oenin	7.16 (±0.08)	5120 (±1050)	0.60

^1^ Antioxidant stoichiometry (number of DPPH radicals reduced per antioxidant molecule). ^2^ Rate constant for the transfer of the first H-atom from antioxidant to DPPH. ^3^ Antioxidant concentration for a doubling of the period of time required for the accumulation of a fixed concentration of polyunsaturated fatty acid (PUFA) hydroperoxides (conjugated dienes).

**Table 2:**
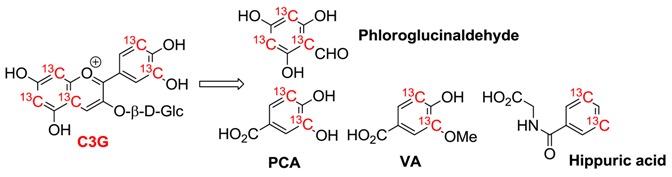
Serum pharmacokinetic profiles of cyanidin 3-glucoside (C3G) and its metabolites in humans after the consumption of 500 mg ^13^C-labelled C3G. From reference [3] (in red is the reference compound and its most abundant metabolites).

Compound	*n*	*C*_max_/nM	*t*_max_/h	*t*_1/2_/h	AUC_0-48_/nM h
Cyanidin-3-glucoside (C3G)	5	141 (±70)	1.8 (±0.2)	0.4	279 (±170)
Protocatechuic acid (PCA)	8	146 (±74)	3.3 (±0.7)	9.9 (±3.4)	1377 (±760)
Phloroglucinaldehyde	4	582 (±536)	2.8 (±1.1)	nd	7882 (±7768)
PCA-sulfates	8	157 (±116)	11.4 (±3.8)	31.9 (±19.1)	1180 (±349)
Vanillic acid (VA)	2	1845 (±838)	12.5 (±11.5)	6.4	23319 (±20650)
VA-sulfates	4	430 (±299)	30.1 (±11.4)	nd	10689 (±7751)
Ferulic acid	7	827 (±371)	8.2 (±4.1)	21.4 (±7.8)	17422 (±11054)
Hippuric acid	8	1962 (±1389)	15.7 (±4.1)	95.6 (±77.8)	46568 (±30311)
